# Comparative in Vitro Study on the Antimicrobial Efficacy of Endodontic Sealers Against Common Oral Pathogens

**DOI:** 10.3390/dj13010017

**Published:** 2024-12-30

**Authors:** Csaba Dudás, Zsuzsanna Bardocz-Veres, Anita Iulia Gyulai, Silvia Izabella Pop, Melinda Székely, Bernadette Kerekes-Máthé, Mónika Kovács

**Affiliations:** 1 Faculty of Dentistry, George Emil Palade University of Medicine, Pharmacy, Science, and Technology of Targu Mures, 38 Gh. Marinescu Str., 540139 Târgu Mureș, Romania; csaba.dudas@umfst.ro (C.D.); gyulai.anita-iulia@stud18.umfst.ro (A.I.G.); 2 Department of Oral Rehabilitation and Occlusology, George Emil Palade University of Medicine, Pharmacy, Science, and Technology of Targu Mures, 38 Gh. Marinescu Str., 540139 Târgu Mureș, Romania; zsuzsanna.bardocz-veres@umfst.ro; 3 Department of Orthodontics, George Emil Palade University of Medicine, Pharmacy, Science, and Technology of Targu Mures, 38 Gh. Marinescu Str., 540139 Târgu Mureș, Romania; 4 Department of Tooth and Dental Arch Morphology, George Emil Palade University of Medicine, Pharmacy, Science, and Technology of Targu Mures, 38 Gh. Marinescu Str., 540139 Târgu Mureș, Romania; melinda.szekely@umfst.ro (M.S.); bernadette.kerekes-mathe@umfst.ro (B.K.-M.); 5 Department of Odontology and Oral Pathology, George Emil Palade University of Medicine, Pharmacy, Science, and Technology of Targu Mures, 38 Gh. Marinescu Str., 540139 Târgu Mureș, Romania; monika.kovacs@umfst.ro

**Keywords:** endodontic sealer, antimicrobial, in vitro study

## Abstract

**Background/Objectives**: Microorganisms are the leading cause of infections in the root canal system, contributing to the failure of endodontic treatments. This in vitro study aimed to compare the antimicrobial effects of four different endodontic sealers: Endomethasone N (Septodont, Saint Maur-des-Fossés, France), Sealapex (Kerr Corporation, Orange, CA, USA), AH Plus Jet (Dentsply DeTrey GmbH, Konstanz, Germany), and MTA Fillapex (Angelus, Londrina, Brazil). **Methods**: The sealers were tested against common oral pathogens, including Enterococcus faecalis, Staphylococcus aureus, Escherichia coli, Candida albicans, and Streptococcus mutans, using the agar diffusion method. Inhibition zones were measured at 24, 48, and 72 h to assess antimicrobial efficacy. **Results**: The results showed that Endomethasone was the most effective sealer against all tested microorganisms, demonstrating consistent inhibition across all time intervals. MTA Fillapex also exhibited a significant antimicrobial effect, particularly against Streptococcus mutans, with its efficacy increasing over time. AH Plus Jet displayed limited effectiveness, showing significant results only against Staphylococcus aureus. **Conclusions:** Overall, this study confirms the superior antimicrobial performance of Endomethasone, while the other materials, particularly MTA Fillapex and Sealapex, also showed notable effects in experimental conditions. The antimicrobial activity of all materials, except AH Plus Jet, increased over the 72-h period.

## 1. Introduction

Microorganisms are widely recognized as the primary cause of infections in the root canal system. Unlike other oral infections, endodontic infections occur in a closed environment, as the root canal is surrounded by hard tissues. These microorganisms play a significant role in most diseases affecting the periapical tissues and dental pulp. When the root canal system is exposed to the oral environment—whether due to injury, decay, or other factors—and the body’s immune response is compromised, infections can develop and rapidly worsen [1,2,3].

Endodontic treatment failure is often caused by microorganisms that persist in the apical portion of the root canal system, even in well-treated teeth. Studies have shown that parts of the root canal space may remain untouched during chemomechanical preparation, allowing bacteria and necrotic tissue to survive, despite an adequate root canal filling radiograph [4,5,6,7]. Environmental factors from the root canal system, such as nutrient availability and the effectiveness of disinfection techniques and preparations, influence the survival of specific pathogens. Disinfectant treatments do not permanently eliminate bacteria in areas such as isthmuses, bifurcations, deltas, lateral canals, and dentinal tubules [8]. Inadequate elimination of microorganisms may contribute to treatment failure. While some bacteria in bifurcations and deltas may have access to nutrients, areas with reduced nutrient availability, like isthmuses and dentinal tubules, are typically destroyed or prevented from migrating into the periapical tissues [9].

Some bacterial species can survive even after long periods of isolation, deriving nutrients from remaining tissues and dead cells. Bacterial growth can be further supported by the leakage of tissue fluids when the root filling fails to seal the dental apex completely. Studies have shown the presence of viable microbial cells in treated teeth with persistent periradicular lesions, suggesting that microorganisms may obtain nutrients from tissue fluids that infiltrate the root canal space [7].

Root canal infections are frequently associated with a diverse range of microorganisms, including *Enterococcus faecalis*, Candida albicans, and various bacterial species, which are known to contribute to endodontic treatment failure [2,10,11]. The persistence of these pathogens highlights the need for effective antimicrobial strategies, particularly in the choice of endodontic sealers. Gutta-percha remains the most widely used core root-filling material today. It cannot bond to dentin in its solid state; this is only possible when it is in a plastic form, which can be achieved through heating or using a solvent. Afterward, gutta-percha may shrink due to cooling or solvent evaporation, often forming gaps. These gaps, together with the accessory canals, are typically filled with sealers. Applying the sealer in a thin layer is recommended, as these materials can also shrink during setting, with shrinkage proportional to their volume [12]. Endodontic sealers are classified mainly according to their setting reaction and composition into several types, including zinc oxide-eugenol, salicylate, fatty acid, glass ionomer, silicone, epoxy resin, tricalcium silicate, and methacrylate resin systems [13].

Recent advancements in dental research have explored antimicrobial additives in endodontics. These studies highlight the potential of incorporating nanoparticles, essential oils, and plant extracts into endodontic sealers and irrigants to improve their antimicrobial efficacy [14,15,16]. Nanoparticles such as silver, zinc oxide, and titanium dioxide have demonstrated broad-spectrum antimicrobial properties, making them promising candidates for combating persistent pathogens [16]. Although the antibacterial effects of vegetable extracts and essential oils are well-documented, their application in endodontics requires further investigation through randomized clinical trials, toxicity assessments, toxicological evaluations, and studies on their interactions with dentin. Additionally, concerns have been raised regarding the physical properties of these materials, particularly their sealing ability. In this case, it fails to perform the tasks mentioned above and may contribute to the failure of the root canal treatment.

This in vitro study aimed to test the null hypothesis that no significant differences exist in the antimicrobial effects of various endodontic sealers. We selected a zinc oxide eugenol-based sealer, an epoxy resin sealer, a calcium hydroxide-containing salicylate, and a tricalcium silicate-based sealer to investigate this.

## 2. Materials and Methods

In this study, the effects of four different types of endodontic sealers: a zinc oxide eugenol-based sealer, Endomethasone N (Septodont, Saint Maur-des-Fossés, France); a calcium hydroxide-containing salicylate, Sealapex (Kerr Corporation, Orange, CA, USA); an epoxy resin-based sealer, AH Plus Jet (Dentsply DeTrey GmbH, Konstanz, Germany); and a tricalcium silicate-based sealer, MTA Fillapex (Angelus, Londrina, Brazil) were evaluated against four bacterial species and one fungal species. The selected microorganisms are commonly found in the oral cavity, necrotic pulp, and various root lesions. Using a custom-made silicone template, standardized samples of the root filling materials were prepared, each with a diameter of 5 mm and a thickness of 2 mm. The sample size calculation was performed with a statistical power of 0.8 and a significance level of 5% using G*Power 3.1.9.7 software (Düsseldorf, Germany). A total of 120 samples were produced, 30 specimens for each tested root canal filling material. Once the sealers had been set, samples were placed on each culture medium and arranged equidistantly.

The fungus was cultured on Sabouraud dextrose agar, Streptococcus on blood agar, and the other microorganisms on plain nutrient agar. After inoculating the bacteria onto the plates, the sealer discs were immediately placed on top. The plates were stored in a thermostat at 37 °C. The sensitivity of the sealers to bacteria and fungi was assessed using the agar diffusion method. Inhibition zones were measured after 24, 48, and 72 h with an inhibition zone ruler specially designed for this purpose. Between measurements, the samples were also stored at 37 °C. The sensitivity of the following strains was tested: *Enterococcus faecalis*, *Staphylococcus aureus*, *Escherichia coli*, *Candida albicans*, and *Streptococcus mutans*.

The values of the inhibition zones were entered into a spreadsheet, where the mean values and standard deviation were calculated. Using GraphPad Prism 10.2.0 (GraphPad Software, Inc., La Jolla, CA, USA), a multifactorial ANOVA test and a post hoc Tukey test were performed. First, we compared the four sealers across the three time intervals, followed by an analysis based on microorganisms.

## 3. Results

**Figure 1:**
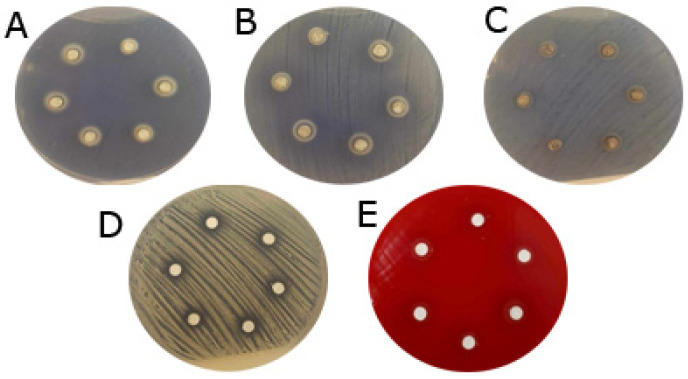
Inhibition zones of Endomethasone samples around *Enterococcus faecalis* (**A**), *Staphylococcus aureus* (**B**), *Escherichia coli* (**C**), *Candida albicans* (**D**), *Streptococcus mutans* (**E**) on prepared agar plates after 24 h.

According to our results, the inhibition zones of Endomethasone (*p* = 0.22) and MTA Fillapex (*p* = 0.001) increased significantly during the 72 h of antimicrobial analysis. The other investigated endodontic sealers showed no significant differences in this regard (*p* > 0.05). Clearly measurable inhibition zones were observed around the Endomethasone samples in all cases. For MTA Fillapex, the most significant inhibition zones were observed around *Streptococcus mutans*, already visible after 24 h (Figure 1). The antimicrobial effect of MTA Fillapex increased across all three investigated time intervals.

The results of the first 24 h are shown in Table 1. The smallest bacterial inhibition zones were observed when the AH Plus Jet endodontic sealer was used. These values were significantly lower compared to Endomethasone (*p* = 0.00006), Sealapex (*p* = 0.004), and MTA Fillapex (*p* = 0.002). AH Plus samples had a significant effect only on *Staphylococcus aureus* (*p* = 0.01).

After 48 h (Table 2), AH Plus Jet showed significantly lower values compared to the other materials (*p* < 0.05). Additionally, the difference in inhibition between Endomethasone and Sealapex was also significant (*p* = 0.0003).

After 72 h (Table 3), AH Plus Jet consistently presented significantly low values compared to all the investigated endodontic sealers (*p* < 0.05). Additionally, the differences between Sealapex and MTA Fillapex were also significant (*p* = 0.0001). The inhibition zones around Sealapex for the *Escherichia coli* strain increased only after 72 h, with no changes between 24 and 48 h. The increasing antimicrobial effect of Sealapex on *Candida* was detectable only after 72 h. After the 72-h time interval, Endomethasone was the most effective against *Enterococcus faecalis*, *Escherichia coli*, and *Streptococcus mutans* strains. At the same time, Sealapex paste was most effective against *Candida albicans*, and MTA Fillapex root filling material showed the greatest efficacy against the *Staphylococcus aureus* and *Streptococcus mutans* strains.

## 4. Discussion

Antimicrobial activity can be provided directly by the sealer itself or indirectly by the entombment of bacteria. This latter can be achieved through a well-executed root canal filling, which prevents bacteria from accessing the periradicular tissues. It is particularly relevant for bacteria on the root canal walls or within the dentinal tubules. On the other hand, bacteria residing in the apical portion of the root canal, including apical deltas and lateral canals, can sustain a persistent infection [17]. For this reason, the antimicrobial effect of a root canal sealer is majorly important. The sealers tested for their antimicrobial effect were the commonly used zinc oxide-eugenol-based sealer (Endomethasone N), a calcium hydroxide-containing sealer (Sealapex), a resin-based sealer (AH Plus Jet) and a calcium silicate-based sealer (MTA Fillapex).

The agar diffusion test is the most commonly used method to determine the antimicrobial efficacy of root canal sealers. This method allows direct comparison of endodontic sealers against the microbe and indicates which material has the maximum antimicrobial activity. The disadvantages of this method are influenced by the diffusibility of root canal sealer across the medium, and the more diffusible materials might show a larger zone of inhibition and antimicrobial activity. Meanwhile, the agar diffusion test cannot differentiate between the sealer’s bactericidal and bacteriostatic effects. Other impediments may vary the results, such as incubation time, sealer/agar contact, and inoculum size. However, reproducible results may be obtained when these drawbacks are precisely controlled. The agar diffusion test may be considered a valid method to evaluate the antimicrobial efficacy of root canal sealers [18].

The selected microorganisms for the present study were *Enterococcus faecalis*, *Staphylococcus aureus*, *Escheria coli*, *Streptococcus mutans*, and a fungus, *Candida albicans*, which may be present in endodontic infections. Several studies have reported that *Enterococcus faecalis* has a high capacity to form biofilm [19] and is considered the most resistant [20]. This microorganism is capable of surviving to the alkaline pH of calcium hydroxide. The antimicrobial effect of resin-based sealers might be attributed to the presence of epoxy resin and amine ingredients.

Huang et al. examined the antimicrobial effects of GuttaFlow2, AH Plus, ProRoot MTA, and RealSeal against *Enterococcus faecalis*, *Escherichia coli*, and *Candida albicans*. In their experiment, culture mediums were assessed using an agar diffusion test from the immediate application of freshly mixed sealers for up to 7 days. Their findings indicated that AH Plus exhibited the strongest initial effect among the mentioned products, which diminished significantly over time. This reduction was not observed in our study, as the starting point of investigation was 24 h after application, rather than immediately after mixing. However, their study also demonstrated the late-stage weakness in antimicrobial efficacy [21]. The low inhibition zone diameters (less than 1 mm) observed for the AH Plus Jet sealer may differ from the results of others due to the difference in study design [22,23]. The underlying reasons may be multifactorial, ranging from the resin-based composition of the sealer to the inherent limitations of the agar diffusion test, as discussed in the limitations section of this study. Notably, authors have attempted to improve the antimicrobial properties of AH Plus by incorporating chlorhexidine (CHX) and cetrimide (CTR). Their findings indicate that adding CHX, CTR, or a combination of both significantly enhances this material’s bactericidal and anti-biofilm capacities [24].

MTA also showed a strong effect in several authors’ experiments, similar to our results [21,25,26]. Tricalcium silicate-based sealers raise the local pH by releasing calcium and hydroxide ions, which enhances their antimicrobial effect [27].

Gomes et al. investigated the antimicrobial effects of Endo Fill, Endomethasone, Endomethasone N, Sealer 26, and AH-Plus against *Candida albicans*, *Staphylococcus aureus*, *Enterococcus faecalis*, *Streptococcus sanguis*, and *Actinomyces naeslundii*. Inhibition zones were measured at 24 and 48 h and again after 7 days. Endomethasone was identified as the most effective sealer in their study, a finding consistent with the results of this paper [28]. A possible reason could be that zinc oxide is a well-documented antimicrobial material due to its ability to generate reactive oxygen species and disrupt bacterial membrane proteins [29]. Several authors have published on the good antimicrobial effects of these endodontic sealers [30,31,32,33].

Our study revealed that Sealapex was the second most effective of the tested materials against *E. faecalis*. The antimicrobial properties of this sealer are influenced by its solubility and the release of calcium ions. At the same time, Sealapex had the strongest effect on *Candida albicans* after 72 h and presented antimicrobial activity against all strains. Similar findings regarding this sealer have been reported in previous studies [34,35,36]. The *Candida* species were detected in low prevalence from intraradicular infections [37]. Some molecular studies reveal that fungi are present in a much higher proportion of primary infections than previously reported [38,39]. The *Candida* species have been detected in root canal-treated teeth in up to 18% of cases in a study conducted by Pinheiro et al. [37]. Fungi invade and infect the root canal during endodontic treatment or inefficient intracanal antimicrobial procedures. *Candida albicans* is the most frequently detected fungal species in root-filled teeth. This species can penetrate the tubular structure of dentine and colonize within the root canal system [38,40,41,42].

Antimicrobial properties are not the only requirement of an ideal endodontic sealer. It should also exhibit strong adhesion to ensure adequate bonding between dentin and root-filling materials. The root-filling material must not shrink during placement or dissolve when exposed to tissue fluids. Additionally, it should resist causing tooth discoloration and maintain reliable bonding, even in moisture. It should be biocompatible, causing no harm to the patient or treatment staff, and arrive in a sterile state or easily sterilizable. The endodontic sealer should not irritate periapical tissues or damage tooth structure while offering antimicrobial or bacteriostatic effects. Furthermore, it should promote the healing of periapical tissues and be radiopaque to allow for more effortless follow-up. It must be set within an appropriate timeframe and provide a tight seal. Ideally, the material should also be easy to mix, apply, and introduce into the root canal, with the option to be easily removed or dissolved if necessary [39,43].

Future studies should include the evaluation of the mechanical properties of the sealers, and newly developed sealers should also be included. The chemical composition of a sealer is essential in terms of the success of the root canal treatment. Research groups are developing and evaluating new and improved materials for different clinical areas, including endodontics [44,45]. Recent studies show that bioceramic sealers supplemented with silver nanoparticles could increase the antimicrobial activity against endodontic pathogens like *E. faecalis* without affecting the mechanical properties of the sealer [46]. Kharouf N. et al. also studied a novel sealer, the BioRoot Flow, which might perform well as a root canal sealer [47].

A strength of this study might be that a range of commonly encountered microorganisms, both bacterial and fungal species, were involved. This variety of pathogens may contribute to the generalizability of the findings. The inhibition zones have been measured at multiple time points (24, 48, and 72 h). This temporal analysis reveals how the sealers perform over time and may provide insights into their sustained antimicrobial properties. Conducting a sample size calculation with a statistical power of 0.8 adds robustness to the experimental design. Finally, the study examines sealers commonly used in current clinical practice in Romania.

The present study has several limitations. In vitro conditions do not fully represent clinical situations. The volume of the sealer and the contact area may differ from the clinical applications. Sealers are applied in the root canal and have a limited contact area with the periradicular tissue. Also, the agar diffusion method’s disadvantages might be considered limitations. This method does not distinguish between microbiostatic and microbicidal properties, and the antimicrobial results are influenced by the solubility and diffusibility of the sealer in the medium [24]. The diffusibility of the root canal sealer across the medium, the incubation time, and the sealer-agar contact might influence the observed inhibition zones. Future studies should also consider assessing the antimicrobial activity in a liquid nutrient medium.

Furthermore, the study design did not include a control group to assess the background antimicrobial activity of the culture media. Considering these limitations, future studies on the antimicrobial effect of different types of sealers using a direct contact test method (DCT) are needed to advance sealers and endodontic treatment techniques [13]. Additionally, 3-dimensional image analysis of live/dead-stained biofilms is planned to provide a more comprehensive understanding of the antimicrobial properties of endodontic sealers with clinical relevance [48].

## 5. Conclusions

The null hypothesis formulated was rejected. Within the limitations of this in vitro study, the results suggest that Endomethasone, Sealapex, and MTA Fillapex endodontic sealers exhibit both anti-bacterial and anti-fungal effects. AH Plus Jet seems to be effective only against *Staphylococcus aureus*. Endomethasone proved to be the most effective against all tested microorganisms. It was statistically proven that the antimicrobial effect of the endodontic materials increased over the first 72 h in experimental conditions; however, this increase was both material- and microorganism-dependent. Further studies are planned to address the limitations highlighted in this study.

## Figures and Tables

**Table 1:** Summary of mean values (mm) and standard deviation (SD) of the bacterial inhibition zones for each sealer after 24 h.

Endodontic Sealers/Oral Pathogens	Endomethasone (±SD)	Sealapex (±SD)	AH Plus Jet (±SD)	MTA Fillapex (±SD)	*p*-Value
*Enterococcus faecalis*	15.25 (±0.66)	13 (±0.89)	0.06 (±0.08)	8.46 (±0.64)	<0.00001
*Staphylococcus aureus*	8.60 (±0.81)	5.88 (±0.66)	6.25 (±0.74)	7.96 (±0.68)	<0.00001
*Escherichia coli*	14.68 (±1.11)	7.16 (±0.76)	0.03 (±0.07)	7.78 (±1.10)	<0.00001
*Candida albicans*	12.83 (±0.98)	6.86 (±1.02)	0.06 (±0.12)	7.81 (±0.78)	<0.00001
*Streptococcus mutans*	7.93 (±0.76)	7.96 (±0.63)	0.03 (±0.08)	10.25 (±0.77)	<0.00001

**Table 2:** Summary of mean values (mm) and standard deviation (SD) of the bacterial inhibition zones for each sealer after 48 h.

Endodontic Sealers/Oral Pathogens	Endomethasone (±SD)	Sealapex (±SD)	AH Plus Jet (±SD)	MTA Fillapex (±SD)	*p*-Value
*Enterococcus faecalis*	17.81 (±1.07)	13.35 (±0.78)	0.06 (±0.08)	10.46 (±0.64)	<0.00001
*Staphylococcus aureus*	8.60 (±0.81)	7.13 (±0.46)	6.25 (±0.74)	10.03 (±0.66)	<0.00001
*Escherichia coli*	14.68 (±1.11)	7.16 (±0.76)	0.05 (±0.08)	9.46 (±0.83)	<0.00001
*Candida albicans*	15.16 (±0.93)	7.25 (±0.79)	0.06 (±0.12)	9.81 (±0.77)	<0.00001
*Streptococcus mutans*	16.35 (±1.19)	7.96 (±0.63)	0.03 (±0.08)	12.25 (±0.77)	<0.00001

**Table 3:** Summary of mean values (mm) and standard deviation (SD) of the bacterial inhibition zones for each sealer after 72 h.

Endodontic Sealers/Oral Pathogens	Endomethasone (±SD)	Sealapex (±SD)	AH Plus Jet (±SD)	MTA Fillapex (±SD)	*p*-Value
*Enterococcus faecalis*	18.20 (±0.73)	13.35 (±0.76)	0.06 (±0.08)	10.71 (±0.59)	<0.00001
*Staphylococcus aureus*	10.95 (±0.85)	7.13 (±0.46)	6.25 (±0.74)	12.63 (±2.14)	<0.00001
*Escherichia coli*	16.65 (±1.24)	9.25 (±0.62)	0.05 (±0.08)	9.75 (±0.55)	<0.00001
*Candida albicans*	18.80 (±0.79)	19.38 (±0.80)	0.06 (±0.12)	11.98 (±0.60)	<0.00001
*Streptococcus mutans*	19.26 (±0.90)	9.93 (±0.55)	0.03 (±0.08)	13.90 (±0.52)	<0.00001

## Data Availability

The data presented in this study are available upon request from the corresponding author.

## References

[1] Saha S., Dhinsa G., Ghoshal U., Afzal Hussain A.F., Nag S., Garg A. (2019). Influence of Plant Extracts Mixed with Endodontic Sealers on the Growth of Oral Pathogens in Root Canal: An in Vitro Study. J. Indian Soc. Pedod. Prev. Dent..

[2] Buonavoglia A., Zamparini F., Lanave G., Pellegrini F., Diakoudi G., Spinelli A., Lucente M.S., Camero M., Vasinioti V.I., Gandolfi M.G. (2023). Endodontic Microbial Communities in Apical Periodontitis. J. Endod..

[3] Siqueira J.F., Rôças I.N. (2022). Present Status and Future Directions: Microbiology of Endodontic Infections. Int. Endod. J..

[4] Zeng C., Hu P., Egan C.P., Bergeron B.E., Tay F., Ma J. (2024). Bacteria debridement efficacy of two sonic root canal irrigant activation systems. J. Dent..

[5] Fornari V.J., Hartmann M.S.M., Vanni J.R., Rodriguez R., Langaro M.C., Pelepenko L.E., Zaia A.A. (2020). Apical root canal cleaning after preparation with endodontic instruments: A randomized trial in vivo analysis. Restor. Dent. Endod..

[6] Neelakantan P., Vishwanath V., Taschieri S., Corbella S. (2022). Present status and future directions: Minimally invasive root canal preparation and periradicular surgery. Int. Endod. J..

[7] Marín-Bauza G.A., Silva-Sousa Y.T.C., da Cunha S.A., Rached-Junior F.J.A., Bonetti-Filho I., Sousa-Neto M.D., Miranda C.E.S. (2012). Physicochemical Properties of Endodontic Sealers of Different Bases. J. Appl. Oral. Sci..

[8] Siqueira J.F., de Uzeda M. (1996). Disinfection by Calcium Hydroxide Pastes of Dentinal Tubules Infected with Two Obligate and One Facultative Anaerobic Bacteria. J. Endod..

[9] Shakya V.K., Gupta P., Tikku A.P., Pathak A.K., Chandra A., Yadav R.K., Bharti R., Singh R.K. (2016). An Invitro Evaluation of Antimicrobial Efficacy and Flow Characteristics for AH Plus, MTA Fillapex, CRCS and Gutta Flow 2 Root Canal Sealer. J. Clin. Diagn. Res..

[10] Ahmed S., Jehad Hassan S., Gajdhar S., Saleh Alhazmi L., Yahya Khalifah R., Alhusain Alrifai J., Salem Aljhdali S., Sheriff Maqbul M. (2024). Prevalence of Enterococcus Faecalis and Candida Albicans in Endodontic Retreatment Cases: A Comprehensive Study. Saudi Dent. J..

[11] Prada I., Micó-Muñoz P., Giner-Lluesma T., Micó-Martínez P., Collado-Castellano N., Manzano-Saiz A. (2019). Influence of Microbiology on Endodontic Failure. Lit. Rev. Med. Oral. Patol. Oral. Cir. Bucal.

[12] Dobrzańska J., Dobrzański L.B., Dobrzański L.A., Gołombek K., Dobrzańska-Danikiewicz A.D. (2021). Is Gutta-Percha Still the “Gold Standard” among Filling Materials in Endodontic Treatment?. Processes.

[13] Kombayashi T., Colmenar D., Cvach N., Bhat A., Primus C., Imai Y. (2020). Comprehensive Review of Current Endodontic Sealers. Dent. Mater. J..

[14] Yudaev P.A., Chistyakov E.M. (2024). Progress in dental materials: Application of natural ingredients. Russ. Chem. Rev..

[15] dos Santos D.C., da Silva Barboza A., Schneider L.R., Cuevas-Suárez C.E., Ribeiro J.S., Damian M.F., Campos A.D., Lund R.G. (2021). Antimicrobial and physical properties of experimental endodontic sealers containing vegetable extracts. Sci. Rep..

[16] Rücker V.B., Balbinot G.D.S., Collares F.M., de Araújo Neto V.G., Giannini M., Leitune V.C.B. (2023). Synthesis of silver core-shell nanoparticles and their influence on an experimental resin endodontic sealer: An in Vitro analysis. Int. Endod. J..

[17] Siqueira J.F., Rôças I.N. (2008). Clinical Implications and Microbiology of Bacterial Persistence after Treatment Procedures. J. Endod..

[18] Poggio C., Lombardini M., Colombo M., Dagna A., Saino E., Arciola C.R., Visai L. (2011). Antibacterial Effects of Six Endodontic Sealers. Int. J. Artif. Organs.

[19] Zheng J.-X., Wu Y., Lin Z.-W., Pu Z.-Y., Yao W.-M., Chen Z., Li D.-Y., Deng Q.-W., Qu D., Yu Z.-J. (2017). Characteristics of and Virulence Factors Associated with Biofilm Formation in Clinical Enterococcus Faecalis Isolates in China. Front. Microbiol..

[20] Bodrumlu E., Semiz M. (2006). Antibacterial Activity of a New Endodontic Sealer against Enterococcus Faecalis. J. Can. Dent. Assoc..

[21] Huang Y., Li X., Mandal P., Wu Y., Liu L., Gui H., Liu J. (2019). The in Vitro Antimicrobial Activities of Four Endodontic Sealers. BMC Oral. Health.

[22] Vibha H., Rathod R. (2017). Assessment of antimicrobial efficacy of bioceramic sealer, epiphany self-etch sealer, and AH-Plus sealer against Enterococcus faecalis: An: In vitro: Study. Endodontology.

[23] Tiwari S., Murthy C.S., Usha H.L., Shivekshith A.K., Kumar N.N., Vijayalakshmi L. (2018). A comparative evaluation of antimicrobial efficacy and flow characteristics of two epoxy resin-based sealers-AH plus and Perma Evolution: An: In vitro: Study. J. Conserv. Dent. Endod..

[24] Bailón-Sánchez M.E., Baca P., Ruiz-Linares M., Ferrer-Luque C.M. (2014). Antibacterial and anti-biofilm activity of AH plus with chlorhexidine and cetrimide. J. Endod..

[25] Morgental R.D., Vier-Pelisser F.V., Oliveira S.D., Antunes F.C., Cogo D.M., Kopper P.M.P. (2011). Antibacterial Activity of Two MTA-based Root Canal Sealers. Int. Endod. J..

[26] Primathena I., Nurdin D., Hermawan H., Cahyanto A. (2021). Synthesis, Characterization, and Antibacterial Evaluation of a Cost-Effective Endodontic Sealer Based on Tricalcium Silicate-White Portland Cement. Materials.

[27] Duarte M.A.H., Duarte M.A.H., de Oliveira Demarchi A.C.C., Yamashita J.C., Kuga M.C., Fraga S.D.C. (2003). PH and Calcium Ion Release of 2 Root-End Filling Materials. Oral. Surg. Oral. Med. Oral. Pathol. Oral. Radiol. Endodontol..

[28] Gomes B.P.F.D.A., Pedroso J.A., Jacinto R.C., Vianna M.E., Ferraz C.C.R., Zaia A.A., Souza-Filho F.J.D. (2004). In Vitro Evaluation of the Antimicrobial Activity of Five Root Canal Sealers. Braz. Dent. J..

[29] Banoee M., Seif S., Nazari Z.E., Jafari-Fesharaki P., Shahverdi H.R., Moballegh A., Moghaddam K.M., Shahverdi A.R. (2010). ZnO Nanoparticles Enhanced Antibacterial Activity of Ciprofloxacin against Staphylococcus Aureus and Escherichia Coli. J. Biomed. Mater. Res. B Appl. Biomater..

[30] Dong W., Chen R., Lin Y.-T., Huang Z.-X., Bao G.-J., He X.-Y. (2020). A Novel Zinc Oxide Eugenol Modified by Polyhexamethylene Biguanide: Physical and Antimicrobial Properties. Dent. Mater. J..

[31] Ajisha R., Neetu J., Aggarwal R., Kaur A., Monika C. (2023). Comparing and Evaluating the Antimicrobial Efficacy of Endodontic Root Canal Sealers against Enterococcus Faecalis, Staphylococcus Aureus, and Candida Albicans at 24, 48, and 72 h: An in Vitro Study. Indian. J. Dent. Sci..

[32] Nazemisalman B., Niaz S., Darvish S., Notash A., Ramazani A., Luchian I. (2024). The Antibacterial Properties of a Reinforced Zinc Oxide Eugenol Combined with Cloisite 5A Nanoclay: An In-Vitro Study. J. Funct. Biomater..

[33] Ruiz-Linares M., Fedoseev V., Solana C., Muñoz-Sandoval C., Ferrer-Luque C.M. (2024). Antibiofilm Efficacy of Calcium Silicate-Based Endodontic Sealers. Materials.

[34] Sipert C.R., Hussne R.P., Nishiyama C.K., Torres S.A. (2005). In vitro antimicrobial activity of Fill Canal, Sealapex, Mineral Trioxide Aggregate, Portland cement and EndoRez. Int. Endod. J..

[35] Estrela C., Bammann L.L., Estrela C.R., Silva R.S., Pécora J.D. (2000). Antimicrobial and chemical study of MTA, Portland cement, calcium hydroxide paste, Sealapex and Dycal. Braz. Dent. J..

[36] Tanomaru J.M., Tanomaru-Filho M., Hotta J., Watanabe E., Ito I.Y. (2008). Antimicrobial activity of endodontic sealers based on calcium hydroxide and MTA. Acta Odontol. Latinoam.

[37] Pinheiro E.T., Gomes B.P.F.A., Ferraz C.C.R., Sousa E.L.R., Teixeira F.B., Souza-Filho F.J. (2003). Microorganisms from Canals of Root-Filled Teeth with Periapical Lesions. Int. Endod. J..

[38] Siqueira J.F., Rôças I.N., Lopes H.P., Elias C.N., de Uzeda M. (2002). Fungal Infection of the Radicular Dentin. J. Endod..

[39] Ordinola-Zapata R., Noblett W.C., Perez-Ron A., Ye Z., Vera J. (2022). Present Status and Future Directions of Intracanal Medicaments. Int. Endod. J..

[40] Siqueira J.F., Rôças I.N., Lopes H.P. (2002). Patterns of Microbial Colonization in Primary Root Canal Infections. Oral. Surg. Oral. Med. Oral. Pathol. Oral. Radiol. Endod..

[41] Baumgartner J., Watts C., Xia T. (2000). Occurrence of Candida Albicans in Infections of Endodontic Origin. J. Endod..

[42] Persoon I.F., Buijs M.J., Özok A.R., Crielaard W., Krom B.P., Zaura E., Brandt B.W. (2017). The Mycobiome of Root Canal Infections Is Correlated to the Bacteriome. Clin. Oral. Investig..

[43] Vishwanath V., Rao H.M. (2019). Gutta-Percha in Endodontics—A Comprehensive Review of Material Science. J. Conserv. Dent..

[44] Paradowska-Stolarz A., Wieckiewicz M., Owczarek A., Wezgowiec J. (2021). Natural Polymers for the Maintenance of Oral Health: Review of Recent Advances and Perspectives. Int. J. Mol. Sci..

[45] Xiao S., Sun G., Huang S., Lin C., Li Y. (2024). Nanoarchitectonics-Based Materials as a Promising Strategy in the Treatment of Endodontic Infections. Pharmaceutics.

[46] Navarrete-Olvera K., Niño-Martínez N., De Alba-Montero I., Patiño-Marín N., Ruiz F., Bach H., Martínez-Castañón G.-A. (2024). The Push-Out Bond Strength, Surface Roughness, and Antimicrobial Properties of Endodontic Bioceramic Sealers Supplemented with Silver Nanoparticles. Molecules.

[47] Kharouf N., Cardinali F., Al-Rayesse R., Eid A., Moujaes Z., Nafash M., Jmal H., Addiego F., Haikel Y. (2024). Mechanical and Physicochemical Characteristics of a Novel Premixed Calcium Silicate Sealer. Materials.

[48] Gong S.Q., Huang Z.B., Shi W., Ma B., Tay F.R., Zhou B. (2014). In vitro evaluation of antibacterial effect of AH Plus incorporated with quaternary ammonium epoxy silicate against Enterococcus faecalis. J. Endod..

